# Supplementary material for: FERN – a Java framework for stochastic simulation and evaluation of reaction networks
Source: BMC Bioinformatics. 2008 Aug 29;9:356. doi: 10.1186/1471-2105-9-356 (PMC2553347; doi:10.1186/1471-2105-9-356)
Supplement: Additional file 1 — FERN distribution, Version 1.3. This archive contains the FERN source code and binaries as well as documentation and example models in FernML and SBML. [file 1471-2105-9-356-S1.zip › fern/doc/javadoc/fern/simulation/class-use/Simulator.html]

Uses of Class fern.simulation.Simulator


---


|  |  |  |
| --- | --- | --- |
| **Overview** \| **Package** \| **Class** \| **Use** \| **Tree** \| **Deprecated** \| **Index** \| **Help** | |  |
| PREV   NEXT | **FRAMES**    **NO FRAMES**     **All Classes** |  |


---


## **Uses of Class fern.simulation.Simulator**

| Packages that use Simulator | |
| --- | --- |
| **fern.benchmark** | Provides classes for benchmarking simulations (time benchmarks, histogram distance calculation). |
| **fern.cellDesigner** |  |
| **fern.cellDesigner.ui** |  |
| **fern.cytoscape** | Provides the classes for the cytoscape plugin. |
| **fern.cytoscape.ui** |  |
| **fern.example** | Contains examples and demonstrations of the framework. |
| **fern.network** | Provides general classes and interfaces for storing network data. |
| **fern.network.sbml** | Provides the classes for parsing and using sbml based networks. |
| **fern.simulation.algorithm** | Provides algorithms for stochastic simultion of reaction networks. |
| **fern.simulation.controller** | Provides classes to control the simulations. |
| **fern.simulation.observer** | Provides classes to observe certain aspects of simulations. |

Uses of Simulator in fern.benchmark

| Fields in fern.benchmark declared as Simulator | |
| --- | --- |
| `protected  Simulator[]` | `SimulatorPerformance.simulators`             Contains the `Simulator`s - use this field to attach `Observer`s. |

| Methods in fern.benchmark that return Simulator | |
| --- | --- |
| `Simulator[]` | `SimulatorPerformance.getSimulators()`             Gets the simulators used by this benchmark. |

Uses of Simulator in fern.cellDesigner

| Methods in fern.cellDesigner with parameters of type Simulator | |
| --- | --- |
| `double` | `CellDesignerPropensityCalculator.calculatePropensity(int reaction, AmountManager amount, Simulator sim)` |

Uses of Simulator in fern.cellDesigner.ui

| Methods in fern.cellDesigner.ui that return Simulator | |
| --- | --- |
| `Simulator` | `OverviewPane.getSimulator()` |

Uses of Simulator in fern.cytoscape

Constructors in fern.cytoscape with parameters of type Simulator

`CytoscapeColorChangeObserver(boolean visualize,
boolean showTrendSteps,
Simulator sim,
CytoscapeNetworkWrapper net,
FernVisualStyle style,
String[] trendSpecies,
double time)`

Uses of Simulator in fern.cytoscape.ui

| Methods in fern.cytoscape.ui that return Simulator | |
| --- | --- |
| `Simulator` | `OverviewPane.getSimulator()` |

Uses of Simulator in fern.example

| Methods in fern.example that return Simulator | |
| --- | --- |
| `Simulator` | `HistogramDistanceTestSet.getSimulator()` |

Constructors in fern.example with parameters of type Simulator

`CellGrowthObserver(Simulator sim,
double generationTime,
double timeOffset)`
  
          Creates the observer for given simulator, generation time and a time offset (because
the simulator starts at time 0 and for one example it actually starts at time 1000)

`HistogramDistanceTestSet(Simulator sim,
double eps,
int runs,
double time,
String species)`

Uses of Simulator in fern.network

| Methods in fern.network with parameters of type Simulator | |
| --- | --- |
| `double` | `PropensityCalculator.calculatePropensity(int reaction, AmountManager amount, Simulator sim)`             Calculates the propensity for a reaction given the amounts of the `AmountManager`. |
| `double` | `AbstractKineticConstantPropensityCalculator.calculatePropensity(int reaction, AmountManager amount, Simulator sim)`             Calculates the propensity for `reaction` by the formula h\*c, where c is the kinetic constant for `reaction` and h is the number of distinct molecular reactant combinations for `reaction`. |

Uses of Simulator in fern.network.sbml

| Methods in fern.network.sbml with parameters of type Simulator | |
| --- | --- |
| `double` | `MathTree.calculate(AmountManager amount, Simulator sim)`             Evaluate the MathTree. |
| `double` | `SBMLPropensityCalculator.calculatePropensity(int reaction, AmountManager amount, Simulator sim)` |
| `void` | `SBMLNetwork.registerEvents(Simulator sim)`             Registers the `SBMLEventHandlerObserver` for each event in the sbml file to the Simulator. |
| `void` | `SBMLEventHandlerObserver.setSimulatorAsync(Simulator sim)` |

Constructors in fern.network.sbml with parameters of type Simulator

`SBMLEventHandlerObserver(Simulator sim,
SBMLNetwork net,
org.sbml.libsbml.Event event)`
  
          Creates the observer.

Uses of Simulator in fern.simulation.algorithm

| Subclasses of Simulator in fern.simulation.algorithm | |
| --- | --- |
| `class` | `AbstractBaseTauLeaping`             Base class for all tau leaping procedures (which are different in the methods choosing the timestep candidates for critical and noncritical reactions). |
| `class` | `AbstractTauLeapingPropensityBoundSimulator`             There are some possibilities to bind the expected change of the propensities by a value epsilon in order to fulfill the leap condition. |
| `class` | `GibsonBruckSimulator`             Implementation of the algorithm of Gibson and Bruck. |
| `class` | `GillespieEnhanced`             This is an enhanced version of the original Direct method developed by Gillespie. |
| `class` | `GillespieSimple`             Implementation of Gillespie's Direct method. |
| `class` | `HybridMaximalTimeStep`             This is an modified version of Maximal Time Step Method by Puchalka and Kierzec. |
| `class` | `TauLeapingAbsoluteBoundSimulator`             There are some possibilities to bind the expected change of the propensities by a value epsilon in order to fulfill the leap condition. |
| `class` | `TauLeapingRelativeBoundSimulator`             There are some possibilities to bind the expected change of the propensities by a value epsilon in order to fulfill the leap condition. |
| `class` | `TauLeapingSpeciesPopulationBoundSimulator`             There are some possibilities to bind the expected change of the propensities by a value epsilon in order to fulfill the leap condition. |

Uses of Simulator in fern.simulation.controller

| Methods in fern.simulation.controller with parameters of type Simulator | |
| --- | --- |
| `boolean` | `SimulationController.goOn(Simulator sim)`             Returns whether or not to go on with the given simulation |
| `boolean` | `OrController.goOn(Simulator sim)` |
| `boolean` | `DefaultController.goOn(Simulator sim)` |
| `boolean` | `AndController.goOn(Simulator sim)` |

Uses of Simulator in fern.simulation.observer

| Methods in fern.simulation.observer that return Simulator | |
| --- | --- |
| `Simulator` | `Observer.getSimulator()`             Gets the simulator |

| Methods in fern.simulation.observer with parameters of type Simulator | |
| --- | --- |
| `boolean` | `AmountAtMomentObserver.goOn(Simulator sim)`             Causes the simulator to stop when `theta` has been passed and the amounts have been recorded. |
| `protected  void` | `Observer.setSimulator(Simulator simulator)`             Sets the simulator. |

Constructors in fern.simulation.observer with parameters of type Simulator

`AmountAtMomentObserver(Simulator sim,
double moment,
String... speciesName)`
  
          Creates the observer for the given simulator, the given theta and the given species

`AmountIntervalObserver(Simulator sim,
double interval,
int... species)`
  
          Creates the observer for a given simulator, a given interval and given species indices

`AmountIntervalObserver(Simulator sim,
double interval,
String... speciesName)`
  
          Creates the observer for a given simulator, a given interval and given species names.

`FireTypeObserver(Simulator sim)`
  
          Create the observer for the given simulator.

`InstantOutputObserver(Simulator sim,
PrintWriter pw)`
  
          Creates the observer for a given simulator and a given `PrintWriter`

`IntervalObserver(Simulator sim,
double interval,
String[] entityName)`
  
          Create the observer for a given simulator, a given interval, given indices with names.

`LeapObserver(Simulator sim,
String... speciesNames)`
  
          Creates the observer with a given simulator and given species

`MultiAmountIntervalObserver(Simulator sim,
String presentationName,
double interval,
int... species)`
  
          Creates the observer for a given simulator, a given interval and given species indices

`MultiAmountIntervalObserver(Simulator sim,
String presentationName,
double interval,
String... speciesName)`
  
          Creates the observer for a given simulator, a given interval and given species names.

`Observer(Simulator sim)`
  
          Creates an observer dedicated to one simulator.

`RandomNumberGeneratorCallObserver(Simulator sim)`
  
          Creates the observer for the given simulator.

`ReactionIntervalObserver(Simulator sim,
double interval,
int... reactions)`
  
          Creates the observer for a given simulator, a given interval and given reaction indices.

`ReactionIntervalObserver(Simulator sim,
double interval,
String... speciesNames)`
  
          Creates the observer for a given simulator, a given interval and given neighbors.

`TriggerObserver(Simulator sim)`

---


|  |  |  |
| --- | --- | --- |
| **Overview** \| **Package** \| **Class** \| **Use** \| **Tree** \| **Deprecated** \| **Index** \| **Help** | |  |
| PREV   NEXT | **FRAMES**    **NO FRAMES**     **All Classes** |  |


---
